# Human intelectin-1 (ITLN1) genetic variation and intestinal expression

**DOI:** 10.1038/s41598-021-92198-9

**Published:** 2021-06-18

**Authors:** Eric B. Nonnecke, Patricia A. Castillo, Amanda E. Dugan, Faisal Almalki, Mark A. Underwood, Carol A. De La Motte, Weirong Yuan, Wuyuan Lu, Bo Shen, Malin E. V. Johansson, Laura L. Kiessling, Edward J. Hollox, Bo Lönnerdal, Charles L. Bevins

**Affiliations:** 1 Department of Microbiology and Immunology, School of Medicine, University of California, Davis, Davis, CA 95616 USA; 2 Department of Chemistry, Massachusetts Institute of Technology, Cambridge, MA 02139 USA; 3 Department of Genetics and Genome Biology, University of Leicester, Leicester, UK; 4 Department of Pediatrics, School of Medicine, University of California, Davis, Sacramento, CA 95817 USA; 5 Department of Inflammation and Immunity, Lerner Research Institute, Cleveland Clinic Foundation, Cleveland, OH 44195 USA; 6 Institute of Human Virology, Department of Biochemistry and Molecular Biology, University of Maryland School of Medicine, Baltimore, MD 21201 USA; 7 Department of Gastroenterology, Hepatology and Nutrition, Digestive Diseases and Surgery Institute, Cleveland Clinic, Cleveland, OH 44195 USA; 8 Institute of Biomedicine, Department of Medical Biochemistry and Cell Biology, University of Gothenburg, Gothenburg, Sweden; 9 The Broad Institute of MIT and Harvard, Cambridge, MA 02142 USA; 10 Koch Institute for Integrative Cancer Research, Massachusetts Institute of Technology, Cambridge, MA 02139 USA; 11 Department of Nutrition, University of California, Davis, Davis, CA 95616 USA; 12 Present Address: Elanco Animal Health, Fort Dodge, IA 50501 USA; 13 Present Address: Medical Laboratories Technology Department, College of Applied Medical Sciences, Taibah University, Almadinah Almunwarah, Saudi Arabia; 14 Present Address: Fudan University, Shanghai, China; 15 Present Address: Department of Surgery, Columbia University Vagelos College of Physicians and Surgeons, New York, NY 10032 USA

**Keywords:** Gastroenterology, Molecular medicine, Lectins

## Abstract

Intelectins are ancient carbohydrate binding proteins, spanning chordate evolution and implicated in multiple human diseases. Previous GWAS have linked SNPs in *ITLN1* (also known as omentin) with susceptibility to Crohn's disease (CD); however, analysis of possible functional significance of SNPs at this locus is lacking. Using the Ensembl database, pairwise linkage disequilibrium (LD) analyses indicated that several disease-associated SNPs at the *ITLN1* locus, including SNPs in *CD244* and *Ly9,* were in LD. The alleles comprising the risk haplotype are the major alleles in European (67%), but minor alleles in African superpopulations. Neither ITLN1 mRNA nor protein abundance in intestinal tissue, which we confirm as goblet-cell derived, was altered in the CD samples overall nor when samples were analyzed according to genotype. Moreover, the missense variant V109D does not influence ITLN1 glycan binding to the glycan β-D-galactofuranose or protein–protein oligomerization. Taken together, our data are an important step in defining the role(s) of the CD-risk haplotype by determining that risk is unlikely to be due to changes in ITLN1 carbohydrate recognition, protein oligomerization, or expression levels in intestinal mucosa. Our findings suggest that the relationship between the genomic data and disease arises from changes in CD244 or Ly9 biology, differences in ITLN1 expression in other tissues, or an alteration in ITLN1 interaction with other proteins.

## Introduction

Intelectins are ancient proteins, spanning chordate evolution and have been implicated in various human disease states, including inflammatory bowel disease (IBD), obesity, non-insulin dependent diabetes mellitus, and asthma^1–3^. Intelectins are multimeric, secreted proteins that exhibit calcium-dependent carbohydrate binding, and each monomer is comprised of a fibrinogen-related domain and C-terminal glycan-binding domain^4–6^. Both preservation across evolution and abundant expression at mucosal surfaces suggest important function of these lectins in innate immunity^4^. Mammalian intelectins can be broadly grouped into ITLN1-like and ITLN2-like proteins based on distinctive N-terminal primary sequence and amino acid length (~ 313 versus ~ 325)^4,7^. The first mammalian intelectin identified (*Itln1*) was found to be expressed in Paneth cells of mice^8^. Some strains of mice have an expanded intelectin locus of up to six *Itln1*-like genes but lack clear *ITLN2*-like orthologs, whereas humans encode a single *ITLN1* gene and a single *ITLN2* gene^7,9,10^. Human ITLN1 is expressed in the small and large intestine, as well as in extra-intestinal tissues, including visceral adipose where the alternative name “omentin” is sometimes used^6,8,11–13^. Orthologs of human *ITLN1* are induced during intestinal parasitemia and related Th2-type immune responses, including asthma^9,14–18^.

Human ITLN1 was found to recognize β-D-galactofuranose, a galactose isomer incorporated into the glycans of microorganisms, including protozoa, fungi, and bacteria, but not mammalian cells^6,19,20^. Within β-D-galactofuranose, ITLN1 binds to the exocyclic vicinal 1,2-diol, a chemical moiety present on other microbial carbohydrate-containing structures, such as Kdo_2_ (Di[3-deoxy-D-manno-octulosonic acid]) of lipopolysaccharide^6,20–22^. This carbohydrate specificity suggests microbial pattern recognition binding for ITLN1, in its otherwise elusive role in innate immunity^6,19,20^.

Genome-wide association studies (GWAS) have identified several single nucleotide polymorphisms (SNPs) present at the *ITLN1* locus as risk alleles for Crohn’s disease, a main subtype of IBD, which is characterized by dysbiosis, epithelial barrier dysfunction, and immune function perturbations; processes consistent with an innate immune dysfunction^1,23–28^. Herein, we further characterize the *ITLN1* locus in the context of IBD-associated SNPs, examine the intestinal expression and glycan binding of ITLN1 associated with risk and non-risk alleles, and localize ITLN1 protein expression to goblet cells of the human small and large intestine.

## Results

### The intelectin-1 locus and Crohn’s disease

To gain insight into the potential influence of IBD-associated allelic variants on expression and protein function, we genotyped surgical specimens from a previously reported cohort of individuals requiring surgical resection for Crohn’s disease or ulcerative colitis (n = 134)^29^. Accordingly, we found by direct sequence analysis of PCR products that the GWAS-identified SNP, rs2274910 (T/C), located in intron 3 of *ITLN1*, was associated with rs2274907 (A/T), which encodes a missense variant in exon 4, where valine is substituted with aspartic acid at amino acid position 109 (V109D)^1,30^. Utilizing the 1000 Genomes Project Phase 3 of the Ensembl database we found rs2274910 and rs2274907 are in linkage disequilibrium (LD) across human populations, consistent with findings of association in our U.S. cohort (Supplemental Table 1)^31,32^. We sequenced the entire coding region of *ITLN1* cDNA in all specimens of this cohort, exploring the potential for additional exon variants in IBD subpopulations. We found rs2274908 (C/T: H89H/Q) exclusively as a synonymous (silent) variant associated with rs2274907^30^. No additional missense variants were identified.

**Table 1:** European and African superpopulation haplotype analysis at the *ITLN1* locus.

	rs4656940 (A/G)	rs11265501 (G/A)	rs12058717 (C/T)	rs11578770 (C/T)	rs4656953 (C/T)	rs1333062 (T/G)	rs2274907 (A/T)	rs2274908 (G/A)	rs2274910 (T/C)	rs2297559 (G/A)	rs4656958 (A/G)
**Location:** Chromosome 1q23.3; GRCh38:CM000663.2 (Forward strand)	(*CD244*) 160,860,478	3’ Intergenic 160,867,138	3’ Intergenic 160,872,059	3’ Intergenic 160,875,846	3’ Intergenic 160,876,052	3’ Intergenic 160,876,494	*ITLN1* Exon 4 160,882,036	*ITLN1* Exon 4 160,882,104	*ITLN1* Intron 3 160,882,256	*ITLN1* Intron 2 160,884,736	5’ Intergenic 160,887,174
**Regional feature**	–	–	–	Putative Regulatory Region	Putative Regulatory Region	Putative Regulatory Region	Coding (V109D)	Coding (H86H/Q)	–	–	Putative Regulatory Region
**Distance** (nucleotides) to rs2274907 (V109D) [Allele correlated with rs2274907 (T)]	−21,558 [A]	−14,898 [G]	−9977 [C]	−6119 [C]	−5984 [T]	−5542 [G]	0	+ 68 [A]	+ 220 [C]	+ 2700 [A]	+ 5138 [G]
**European** LD: rs2274907 D’, r^2^ [AF]	0.83–0.95, 0.28–0.46 [G, 19.0]	0.84–0.95, 0.33–0.47 [A, 20.0]	Abs [T, 0.0]	1.00, 0.17–0.36 [T, 10.0]	1.00, 1.00 [T, 68.0]	1.00, 1.00 [G, 68.0]	[T, 68.0]	1.00, 1.00 [A, 68.0]	1.00, 1.00 [C, 68.0]	0.97–1.00, 0.87–0.98 [A, 68.0]	1.00, 0.97–1.00 [G, 68.0]
**African** LD: rs2274907 D’, r^2^ [AF]	0.41–0.83, 0.05–0.27 [G, 40.0]	0.89–1.00, 0.65–0.78 [A, 63.0]	0.71–1.00, 0.11–0.17 [T, 32.0]	Abs [T, 0.0]	0.97–1.00, 0.62–0.81 [T, 26.0]	0.92–1.00, 0.78–0.96 [G, 32.0]	[T, 33.0]	1.00, 0.87–1.00 [A, 31.0]	1.00, 0.68–0.87 [A, 28.0]	0.50–0.91, 0.09–0.36 [A, 55.0]	0.43–0.81, 0.07–0.31 [G, 54.0]

LD, linkage disequilibrium; AF, allele frequency; Abs, absent. D’ and r^2^ values represent co-inheritance range (i.e., lowest and highest values) for each sub-population within African and European superpopulations^32,35^.

**Figure 1:**
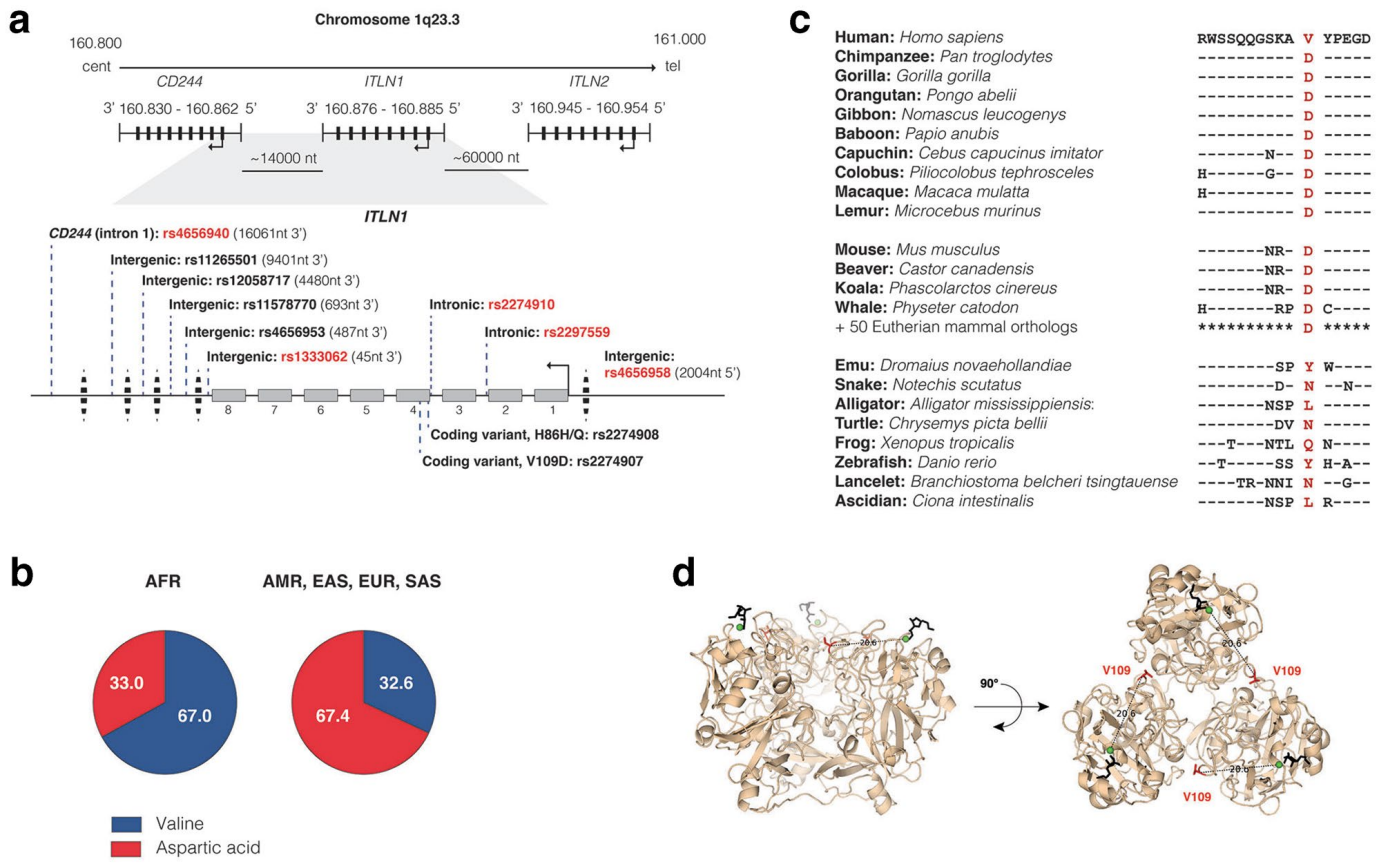
*ITLN1* polymorphisms. (**a**) The physical arrangement of *ITLN1* relative to centromere/telomere orientation and flanking genes on chromosome 1q23.3 is diagramed, along with single nucleotide polymorphisms associated with human disease, including IBD: rs4656940^23^, rs11265501^58^, rs12058717^59^, rs11578770^60^, rs4656953 ^61^, rs1333062^24^, rs2274907^30^, rs2274908^30^, rs2274910^1^, rs2297559^25^, and rs4656958^25–27^. Red text highlights GWAS-identified SNPs. (**b**) 1000 Genomes Project Phase 3 super population allele frequency (percentages) of rs2274907 (A/T, V109D): AFR, African; AMR, American, EAS, East Asian; EUR, European; SAS, South Asian^31,32^. (**c**) 3D structure of human trimeric ITLN1 bound to β-D-galactofuranose (black). The distance between V109 residue (red) and coordinating calcium (green) are indicated on each monomer (PDB: 4WMY). (**d**) Amino acid sequence adjacent to coding variant rs2274907 (V109D) in orthologs of human ITLN1 (“—” identical residue present ITLN1).

To display allelic variants associated with disease, we constructed a locus map of *ITLN1* (Fig. 1A) annotated with published SNPs as outlined by the National Human Genome Research Institute—European Bioinformatics Institute (NHGRI-EBI) Catalog and NCBI-LitVar database, including variants within *ITLN1* and its flanking regions intergenic to *CD244* (3’) and *ITLN2* (5’)^33,34^. *ITLN1* and *CD244* (rs1333062 and rs4656940, respectively) have been annotated together as risk loci for IBD, due in part to their close proximity (~ 14,000 nt)^23,24^. The SNP rs133062 is located in the 3’-flanking region of *ITLN1*, 46 nt intergenic to exon 8, where rs4656940 is located within intron 1 of *CD244*. The odds ratio (OR) for Crohn’s disease of rs4656940 is approximately equivalent (1.15; 95% confidence interval [CI] = 1.09–1.21) to that of rs2274910 (1.14; 95% CI = NR) of *ITLN1*^1,23^. Several annotated SNPs in *CD244* (rs4656942 and rs11265498) and in the adjacent gene *LY9* (rs540254 and rs560681), but not any in neighboring *SLAMF7*, are in LD with GWAS-identified rs2274910 and rs4656940 (Supplemental Table 2)^35^. In addition, rs4656958 located in the 5' flanking region of *ITLN1* (2004 nt 5’ to exon 1) has also been identified by GWAS as a risk allele for IBD (OR = 1.06; 95% CI = 1.03–1.09)^25–27^. Thus, GWAS-identified IBD-associated SNPs are located within *ITLN1* and its flanking regions*,* and these SNPs are in LD with SNPs in *CD244* and *Ly9*, which are genes located 3’ to *ITLN1*. Data in the GTEx browser revealed that quantitative trait loci (QTL) for expression (eQTL) in blood for *ITLN1* and *CD244* are both affected by several disease risk SNPs, including rs1333062, rs2274907 and rs2297559 (Supplemental Table 3)^36^. In addition, QTL for splicing (sQTL) of *USF1* was similarly affected by these SNPs, as was sQTL for *Ly9* by rs465940. No significant eQTLs or sQTLs were identified in small intestine or colon tissue^36^. Although other genes located at 1q23.3 may be responsible for the disease association, we chose to investigate *ITLN1* as the strongest candidate based on our prior knowledge of the function of intelectins.

Further review of *ITLN1* rs2274910 (C/T) revealed a distinction in the frequency of the GWAS-identified risk allele (C) between African (28%) and non-African (67%) superpopulations (SP)^31,32^. This prompted us to further explore allele frequency in the context of different SP groups. The study populations for most IBD-focused GWAS are comprised primarily of non-African SP, including investigations identifying *ITLN1* as a risk locus^1,23,25–27,37^. Using the Ensembl database, we performed pairwise LD analyses of disease-associated *ITLN1* variants (Fig. 1a, Table 1) in African and European SP using rs2274907 (A/T: V109D) as a focus point simply due to it being a missense variant. In addition to being in complete LD (D’ = 1.0, r^2^ = 1.0) with rs2274910, rs2274907 (V109D) was found also to be in LD with GWAS-identified alleles across the *ITLN1* locus, where risk alleles are the major alleles in European SP and minor alleles in African SP (Table 1, Fig. 1b)^31,32,35^.

The allele genotype frequencies in European SP: VV (7.6%), VD (49.3%), and DD (43.1%) contrasts sharply with that in the African SP VV (45.7%), VD (43.1%), and DD (11.2%) (Supplemental Fig. 1). In our small cohort of surgical specimens, allele frequencies were not significantly different for those diagnosed as either Crohn’s disease or ulcerative colitis as compared to respective controls, but there was a suggestion of skewing in specimens diagnosed as ileal compared to colonic Crohn’s disease, worthy of follow-up analysis in a larger cohort (Supplemental Table 1). Our findings clarify that rs2274907 (T, D109), which is in complete LD with GWAS-identified rs2274910 risk allele (C), is the major allele in non-African SP; in Crohn’s disease multiple GWAS report a further skewing of this high frequency of the risk allele observed in the studied populations^1,23–27^.

### Intestinal intelectin-1 expression, glycan-binding, and oligomerization is unaltered by missense variant rs2274907 (A/T, V109D)

**Figure 2:**
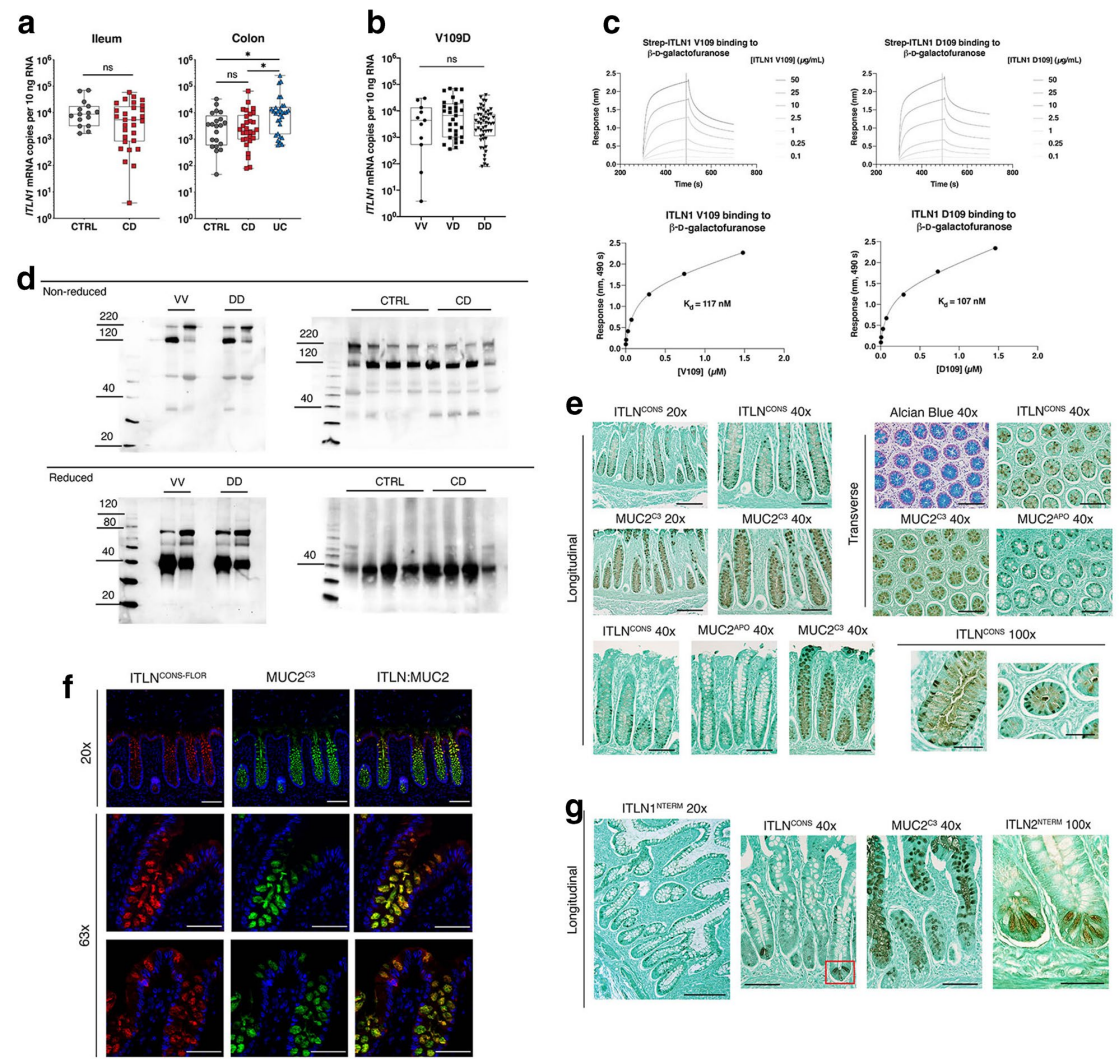
ITLN1 mRNA and protein expression in the small intestine and colon. (**a**) *ITLN1* mRNA expression in surgical specimens of ileum and colon according to diagnosis. The mRNA copy number per 10 ng total RNA was determined with quantitative real-time RT-PCR using external cDNA standards (see methods). (**b**) *ITLN1* mRNA expression by V109D genotype (excluding ulcerative colitis samples). Box plots present the median and first and third quartiles, whiskers present the range. Statistical analysis of *ITLN1* mRNA expression was performed using either a Kruskal–Wallis test with Dunn’s multiple comparisons or two-tailed Mann–Whitney U test; * *p* < 0.05. (**c**) Representative real-time bio-layer interferometry responses of recombinant ITLN1 V109 and D109 binding to immobilized β-D-galactofuranose (upper). Responses at 490 s were plotted against ITLN1concentration and fit to a single site binding equation to determine Kd (lower). Data is representative of three separate experiments. (**d**) SDS-PAGE (4–20% gradient) immunoblots of human ileum by V109D genotype (left, n = 2 per genotype) and disease state (right). (**e**) Immunohistochemistry of human colon using the following antibodies: ITLN conserved (ITLN^CONS^), ITLN1-specific (ITLN1^NTERM^), MUC2 mature (MUC2^C3^), deglycosylated MUC2 (MUC2^APO^, PH1900). Tissue counterstained by DAPI (blue). (**f**) Immunofluorescence of human colon using ITLN^CONS-FLOR^ (anti-chicken; Alexa Flour 647) and MUC2^C3^ (anti-rabbit; Alexa Flour 488). (**g**) Immunohistochemistry of human ileum; ITLN2-specific (ITLN2^NTERM^) antibody. Red box indicates Paneth cells identified with the ITLN^CONS^ antibody. CTRL = control (non-Crohn’s disease and non-ulcerative colitis), CD = Crohn’s disease, UC = ulcerative colitis. Images in (**e**–**g**) are representative of multiple (> 6 fields) from 9 specimens. Light microscopy scale bars: 20x (200 µm), 40x (100 µm), and 100x (50 µm); fluorescence microscopy scale bars: 20x (100 µm) and 63x (50 µm).

Di Narzo, et al. examined blood and intestinal eQTLs from a therapy-resistant Crohn's disease cohort^38^. Their analysis indicated eQTLs for *ITLN1* in intestine (but not blood) are linked to GWAS-identified risk alleles rs4656958 and rs2274910, and eQTLs for *CD244* in blood (but not intestine) are linked to the risk alleles rs4656958, rs4656940 and rs2274910. In both cases, a statistically significant decrease in expression is associated with the GWAS-identified SNPs. To better clarify expression patterns of *ITLN1* within intestinal tissue, we generated an *ITLN1*-specific quantitative real-time PCR assay to enumerate the absolute mRNA transcript levels. *ITLN1* mRNA was abundant in each anatomic segment across both small and large intestine (Supplemental Fig. 2a). Similarly, high levels of *ITLN1* gene expression were observed in surgical specimens of both ileum and colon (Fig. 2a). Crohn’s disease diagnosis did not alter transcript levels in either small or large intestine (Fig. 2a). Of note, *ITLN1* expression in the colon was elevated (*p* < 0.05) in specimens from individuals diagnosed with ulcerative colitis (Fig. 2a). To determine whether *ITLN1* expression is modified by risk-associated haplotype, we stratified the data by genotype. In ileum, colon, and combined sample sets, the risk associated haplotype (queried by rs2274907 genotype) did not modify *ITLN1* expression (Fig. 2b and Supplemental Fig. 2b). Together, these findings suggest that the mucosal transcript level of *ITLN1* is unaltered in Crohn’s disease. Moreover, the risk-associated haplotype does not modify mucosal *ITLN1* expression (Table 1).

The distinguishing chemical properties of valine and aspartic acid provided impetus to further explore the potential of missense variant V109D to modify ITLN1 oligomeric structure or function. Using PyMOL to visualize the published crystal structure of human ITLN1 (PBD: 4WMY), residue 109 was found to be located at the protein surface; however, it is not located at the sites annotated for glycan binding and/or calcium coordination (Fig. 1c)^20,39^. Bio-layer interferometry analysis found that recombinant V109-ITLN1 reversibly bound to immobilized β-D-galactofuranose with kinetics and affinity that mirror recombinant D109-ITLN1 (117 nM versus 107 nM, respectively) (Fig. 2c). Without finding discernable influence on glycan binding, we next tested if the V109D change altered ITLN1 oligomerization state and stability. For other secreted lectins (e.g., ficolins and mannose-binding lectin), oligomerization is important for avidity in binding cell-surface glycans as well as subsequent effector functions^40–42^. Previous studies report that human ITLN1 forms stable homotrimers, consisting of ~ 35 kDa monomers, connected through intermolecular disulfides (C31 and C48)^6,11,43^. Analysis of human ileal lysates confirmed that ITLN1 forms a stable homotrimer at approximately 110 kDa; however, a consistent ITLN1 band near the ~ 220 kDa marker was also identified using a gradient SDS-PAGE (4–20%). This suggests that human ITLN1 forms higher molecular weight oligomers (likely dimers of homotrimers), which are stable under denaturing (but not reducing) conditions (Fig. 2d). These findings agree with those reported for an amphibian ITLN1 ortholog, where dimers of homotrimers were shown to mediate bacterial agglutination^40^. We also performed immunoblots of ileal specimens isolated from V109 and D109 homozygote individuals to determine the potential influence on oligomer formation. Neither VV or DD genotype affected the formation of homotrimers or hexamers (Fig. 2d). These data suggest that that it is unlikely that the role of V109D variation in Crohn’s disease pathogenesis results from alterations in ITLN1 glycan binding or oligomerization.

Alternatively, we reasoned that D109 might influence protein stability, and that such differences would be observed as changes in protein abundance. In human ileal samples, we observed that under reducing conditions ITLN1 was present primarily as a monomer at ~ 40 kDa, with the exception of a minor fraction migrating at ~ 70 kDa (Fig. 2d). This minor band may represent a covalent non-reducible dimer but further study is needed to fully characterize this entity. Human ITLN1 possesses a single glycosylation site at N163, which slightly increases the molecular weight from that predicted (33 kDa) by the deduced amino acid sequence^43^. Ileal samples from individuals diagnosed with Crohn’s disease demonstrated equivalent relative proportions of protein oligomers and no additional degradation products compared to control specimens (Fig. 2d). Thus, ITLN1 was found to be an abundant protein in the small intestine, where expression was comparable in specimens from control and Crohn’s disease biopsies, consistent with mRNA expression data (Fig. 2a, d).

To further investigate the possible significance of V109D variation in Crohn’s disease, we performed a sequence analysis (NCBI BLAST) of the neighboring amino acid region across various ITLN1-like orthologs. In all Eutherian mammals queried (n = 63), aspartic acid (D) was reported as the amino acid at the corresponding position (Fig. 1d). Even amongst primates, valine at position 109 is unique to *Homo sapiens*. Nevertheless, other chordates were found to encode hydrophobic amino acids like valine (e.g., leucine) at this position, including ancient ascidians (Fig. 1d). While aspartic acid marks the risk-allele associated with Crohn's disease, the V109D variation is not a “mutation”, despite its location in a conserved domain of the protein and differences in chemical properties of these residues. Furthermore, V109, or the genetic variants in LD with this allele, could be considered “protective” in the context of Crohn’s disease.

### Human intelectin-1 is a goblet cell product

In mice, where mammalian intelectins were first discovered, *Itln1* is expressed in Paneth cells^8^. In contrast, a recent single-cell RNA sequence analysis investigation identified *ITLN1* mRNA in goblet cells of the ileum and colon^44^. We sought to localize ITLN1 protein in this these tissues to confirm and extend this cell-type expression pattern in humans. Both ITLN1 and ITLN2 are expressed in the human small intestine and cannot be distinguished using most commercially available antibodies; therefore, we generated paralog-specific antibodies to clarify the cellular origin of ITLN1. Colon biopsies of non-IBD subjects, preserved in fixatives to retain mucus (either Carnoy’s fixative or HistoChoice™), were incubated with either antisera targeting a domain highly conserved in intelectins (ITLN^CONS^) or targeting the N-terminal specific domain (ITLN1^NTERM^). Both ITLN^CONS^ and ITLN1^NTERM^ immunoreactivity were localized to colonic goblet cells (Fig. 2e, Supplemental Fig. 2c). To confirm goblet cell localization, samples were stained with a mucin-2 (MUC2) antibody generated against a peptide sequence located within the C-terminal domain (adjacent to the autocatalytic sequence) of the mature, glycosylated MUC2 protein (MUC2^C3^); this antibody targets goblet cell granules and secreted protein^45^. While MUC2^C3^ stained goblet cells most strongly in their secretory granule, the cellular staining pattern of ITLN1 was different. Specifically, punctate staining of ITLN1 adjacent to the granule and relatively weak staining within the granule was a feature of all ITLN1-positive samples tested (n = 6 colon, n = 3 ileum). To further characterize the staining pattern of ITLN1 within goblet cells, we used another MUC2 antibody that targets the tandem repeat region of the apo-MUC2 protein (PH1900, MUC2^APO^). This antibody identifies non-*O*-glycosylated MUC2, which resides within the endoplasmic reticulum region of goblet cells^46,47^. MUC2^APO^ staining of the cytoplasmic region in goblet cells mimicked the staining pattern for ITLN1^NTERM^ (Fig. 2e, Supplemental Fig. 2C). At higher magnification, ITLN1^NTERM^ staining was identified in both cytoplasmic and granule compartments, and readily apparent in both longitudinally and transversely orientated tissue sections (Fig. 2e). To better localize ITLN1 within goblet cells, we generated a chicken anti-ITLN antibody (conserved, ITLN^CONS-FLOR^) for dual labeling with the rabbit-derived MUC2^C3^ reagent in fluorescence microscopy. As observed by conventional microscopy, ITLN1 and MUC2 largely colocalized within colonic goblet cells, where ITLN1 was also found occasionally in the cytosolic compartments adjacent to nuclear (DAPI) staining (Fig. 2f). Immunohistochemistry of human ileum using the ITLN1^NTERM^ antibody also identified goblet cells, where the aforementioned cytosolic and granular staining pattern was also evident (Fig. 2g). It is worth noting that as a feature distinct from findings in mice, human Paneth cells remained unstained with the ITLN1^NTERM^ antibody (Supplemental Fig. 2D). Although staining of human ileum with the ITLN^CONS^ antibody identified Paneth cells in addition to goblet cells, paralog-specific antibodies determined Paneth cell staining to be human ITLN2 (Fig. 2g, and Nonnecke, et al.: unpublished observations). Together, our results demonstrate that human ITLN1 is expressed in goblet cells, both in the ileum and colon.

## Discussion

Various GWAS investigations have identified SNPs at the *ITLN1* locus as risk alleles for Crohn’s disease^1,23–27^. We observed that these SNPs are in LD, forming a risk haplotype that extends into the adjacent *CD244* and *Ly9* genes. Given the abundant expression of ITLN1 in intestinal tissues and the likely role of this lectin in innate immunity, we focused our investigation on ITLN1 in the intestine.

The first GWAS-identified SNP at this locus (rs2274910) is located in intron 3 of *ITLN1*^1^. We determined that this variant was associated with an adjacent SNP in exon 4 (rs2274907), which encodes a valine to aspartic acid amino change at residue 109. Although valine is the most common allele in African SP, aspartic acid is both the most common and the disease-associated allele in European SP—the primary population studied in IBD GWAS^1,23–27^. Our results clarify that V109D is in LD with other GWAS-identified Crohn's disease-associated risk SNPs, resulting in a risk haplotype. Our data suggest that mRNA transcript and protein abundance of mucosal ITLN1, now confirmed to be goblet-cell derived, is unaltered in the Crohn’s disease samples overall, as well as in samples analyzed according to rs2274907 (A/T, V109D) genotype in both disease and non-disease specimens. We clarify that ITLN1 D109 is the ancestral amino acid in mammalian orthologs, supporting that D109 is not a mutation. Furthermore, our data indicate that any role(s) of V109D in Crohn’s disease pathogenesis is unlikely due to changes in ITLN1 carbohydrate recognition or protein oligomerization.

Although multiple GWAS have identified *ITLN1* SNPs in Crohn’s disease, the calculated OR(s) noted above are modest compared to variants of nucleotide-binding oligomerization domain-containing protein 2 (*NOD2/CARD15*): rs2066847 (frameshift variant L1007fsX1008: OR = 3.99; CI = NR) and interleukin 23 receptor (*IL23R*): rs11465804 (intron variant T/G: OR = 1.38—2.56)^1,48–50^. The OR for Crohn’s disease risk associated with the susceptible haplotype should be viewed from the perspective that the risk alleles are also the major alleles in European SP. Unlike *NOD2/CARD15* (rs2066847) and *IL23R* (rs11465804), which are rare variants (1% and 3% globally), *ITLN1* rs2274910 (T/C), and other alleles forming the risk haplotype are common, where T and C alleles of rs2274910 are present at 43% and 57%, respectively, across Phase 3 Individuals. Similarly, the Crohn’s disease risk allele of autophagy-related 16-like 1 (*ATG16L1*) rs2241880 (A/G) is common (60:40%) and associated with a modest OR (1.45; CI = 1.27—1.64)^31,32^.

Further investigation is required to determine the mechanism by which genetic variation at this locus affects risk for Crohn's disease. In this study, we tested relevant possibilities, and our data support that it is unlikely to be due to ITLN1 expression in intestinal goblet cells. It is possible that expression of ITLN1 in another tissue could underlie disease risk. Moreover, expression of other genes at this locus, notably either *CD24*4 or *Ly9*, which include SNPs in LD with the risk haplotype might explain disease risk^33–35^. Based on available QTL data, expression of *USF1* or *CD244* in whole blood would also be reasonable candidates for future investigation^36^.

## Methods

### Human specimens and ethics statement

All methods were carried out in accordance with relevant guidelines and regulations. Surgical specimens were obtained at The Cleveland Clinic Foundation (Cleveland, OH) with informed consent from all patients, and deidentified under protocols approved by the Institutional Review Board at The Cleveland Clinic Foundation as previously described^29^. These specimens were collected from subjects undergoing surgery for Crohn’s disease (CD), ulcerative colitis (UC), or non-CD/UC-related (control, CTRL) surgical indications (i.e., colon cancer, bowel obstruction, and familial adenomatous polyposis). Race was recorded for these specimens based on medical record reporting. Diagnoses were based on standard criteria using clinical, radiological, endoscopic and histopathological findings^29^. Exclusion criteria included the diagnoses of backwash ileitis, indeterminate colitis, concurrent CMV or *C. difficile* infection. Samples were immediately either snap frozen in liquid nitrogen (for RNA, DNA, and protein analysis) or placed into tissue fixative for histology. The mucosa, utilized for mRNA and protein expression, was dissected away from the intestinal/colonic serosa and associated adipose tissue. The overview of regional mRNA expression (Suppl. Figure 1) used commercially-obtained RNAs of human tissue (Biochain, Newark, CA).

### Databases

Single nucleotide polymorphisms (SNPs) referenced to human *ITLN1* were identified using the NHGRI-EBI Catalog of human genome-wide association studies (https://www.ebi.ac.uk/gwas/home) and NCBI-LitVar (https://www.ncbi.nlm.nih.gov/CBBresearch/Lu/Demo/LitVar/#!?query =) databases^33,34^. Further investigation of identified SNPs, including descriptions of putative function and population frequency, was procured from Ensembl and the 1000 Genomes Project (Phase 3)^31,32^. Allele (i.e., nucleotide) associations and linkage disequilibrium were studied using the NCBI-LDlink (LDpair) database (https://ldlink.nci.nih.gov/?tab=help#LDpair)^35^. QTL analysis was queried at The Genotype-Tissue Expression (GTEx) Portal (https://www.gtexportal.org/home/)^36^. Missense variant rs2274907 (V109D) was applied to the referenced crystal structure of human ITLN1 (PBD ID: 4WMQ) using PyMOL software (The PyMOL Molecular Graphics System, Version 1.8.2.1 Schrödinger, LLC., https://pymol.org/2/) to orientate location.

### Genotyping

Genomic DNA was isolated and purified using the QIAamp DNA minikit (Qiagen, Germantown, MD) according to the manufacturer’s protocol. Isolated DNA was quantified by ultraviolet absorbance spectroscopy (260 nm) using a NanoDrop spectrophotometer (Thermo Scientific/NanoDrop Products, Wilmington, DE). Exon variants in each specimen of our IBD cohort were explored by Sanger sequencing of cDNA corresponding to the entire coding region of *ITLN1* amplified using specific primers (2 s: 5’- GGAGCGTTTTTGGAGAAAGCTGCA-3’ and 1147a: 5’- ATTAACATTCTAGCTACTGGGT-3’). The sequenced PCR products were aligned to the NCBI-*ITLN1* reference (NM_017625.3), identifying rs2274907 (A/T: V109D) and rs2274908 (C/T: H89H/Q) in some specimens. Association of single nucleotide polymorphisms rs2274910 (T/C) and rs2274907 (A/T) was identified by PCR amplification from genomic DNA across adjacent regions in multiple samples, including both homozygous, as well as heterozygous (T/C and A/T) genotypes using specific oligonucleotide primers (1062 s: 5’-ACAGGAGCCTTTAGGCCATGT-3’, 1431a: 5’-AGTGGCACCAACCTTGTAGTC-3’). The PCR reactions were initiated by denaturation of the DNA template at 95 °C for 10 min followed by 45 cycles consisting of 95 °C for 15 s, a -1 °C per two-cycle ‘touchdown’ annealing temperature for 5 s (i.e., 65 °C to 58 °C), and 10 s at 72 °C. Primers were designed using MacVector Software (version 16, MacVector, Apex, NC), and synthesized by Invitrogen Life Technologies (Invitrogen, Carlsbad, CA).

### RNA isolation

The general procedures for RNA isolation and synthesis of cDNA were previously described by our group^51,52^. For the current study, RNA was freshly isolated for all specimens from frozen tissue^29^. Briefly, frozen tissue was dispersed using Brinkmann Polytron homogenizer in guanidinium thiocyanate (GTC) buffer (4–5 M GTC, 0.5% sodium-N-lauryl sarcosine, 10 mM EDTA, 20 mM Tris–HCl 0.1 M 2-mercaptoethanol). Homogenates were layered above 5.7 M CsCl/EDTA in diethyl pyrocarbonate (DEPC) H2O in polyallomer tubes (11 × 34 mm; Beckman, Indianapolis, IN). Using a TLS-55 swinging bucket rotor (Beckman, Indianapolis, IN) samples were centrifuged for 3 h at 55,000 rpm in a TL-100 ultracentrifuge (Beckman). Following centrifugation, excess GTC and CsCl were removed by aspiration and the RNA pellet was solubilized with RNA sample buffer (0.5% N-lauroylsarcosine, 5 mM EDTA, 5 mM tris, and 5% 2-mercaptoethanol). The RNA-buffer solution was transferred to tubes containing an equal volume of 50:50 phenol–chloroform (Calbiochem/EMD Millipore, San Diego, CA), vortexed, and centrifuged for 3 min at 13,000 rpm. The resulting supernatant was washed with 100% chloroform (Sigma-Aldrich, St. Louis, MO), centrifuged, and added to tubes containing 100% ETOH and 0.3 M sodium acetate. Following overnight incubation at -80 °C, RNA was precipitated by centrifugation for 20 min at 10,000 rpm and washed once with 80% ETOH. The resulting RNA pellet was re-suspended in DEPC H2O, and concentration was determined by ultraviolet absorbance spectroscopy (260 nm)^51,52^.

### cDNA synthesis

RNA (2–5 μg) was precipitated under centrifugation, washed once with 80% ETOH and re-suspended in DEPC H2O. cDNA synthesis was performed according to the procedures outlined by the SuperScript III First-Strand cDNA Synthesis System (Invitrogen, Thermo Fischer Scientific, Waltham, MA). The cDNA product was purified using a column QIAquick PCR Purification Kit (Qiagen, Germantown, MD).

### Quantitative real time PCR (qRT-PCR)

Primers specific for human *ITLN1* (211 s: 5’-CTTCGTCTCCATCTCTGCCC-3’, 297a: 5’-CTGGTAGATAACACCATTCTC-3’) and *ACTB* (128 s: 5’- TGATGGTGGGCATGGGTCAG-3’, 220a: 5’- CGTGCTCGATGGGGTACTTCAG-3’) were designed using MacVector version 16 Software. Real-time PCR was performed on cDNA template corresponding to 10 ng of RNA using a Roche Diagnostics Lightcycler 2.0 (Roche, Indianapolis, IN) as previously described^51,52^. Briefly, each reaction included 4 mM MgCl2, 0.5 μM forward and reverse primers, and 1 × LightCycler FastStart SYBR Green I mix (Roche, Mannheim, Germany). A control without cDNA template was included in the procedural assay. The PCR conditions were: initial denaturation at 95 °C for 10 min, followed by 45 cycles with each cycle consisting of denaturation, 95 °C for 15 s; annealing at 60 °C for 5 s; and extension at 72 °C for 10 s. Following the cycle runs, samples were denatured to establish the melting temperature(s) of the PCR product. The sample melt temperatures were compared to that of the internal standard to confirm template specificity. Absolute quantification of target gene within each sample was obtained by inclusion of an external cloned cDNA standard of known concentration, where crossing point (i.e., cycle number) is matched to a standard curve. Data was plotted in Prism version 9.0.0 (GraphPad).

### Rabbit anti-ITLN1 polyclonal antibodies

Analysis of the pre-ITLN1 deduced protein sequence using the SignalP4.0 algorithm indicated signal cleavage would occur between G16 and W17^53^. The adjacent 19 amino acids of ITLN1 (WSTDEANTYFKEWTCSSSP) comprised a completely unique sequence compared to the corresponding region of the human ITLN2 sequence (AAASSLEMLSREFETCAFSFS). Adjacent to this discriminating 19-amino acid sequence (W17 to P35) of ITLN1 is a domain beginning at amino acid S36 that has very high sequence identity to ITLN2 as well as other intelectin orthologs. BLASTp search of the non-redundant protein database with the 19-amino acid W17 to P35 peptide sequence yielded numerous primate ITLN1 orthologs with highly similar sequence (> 80%), but no other mammalian proteins. This 19-amino acid peptide was synthetically prepared, purified and characterized to confirm proper mass, as previously described^54^. The peptide was coupled to ovalbumin (OVA, Thermo Fischer Scientific, Waltham, MA) at a ratio of 20 molecules peptide per molecule OVA using two strategies. First, one equivalent volume of 0.2% glutaraldehyde was added to the 20:1 peptide/ovalbumin mixture in PBS; the mixture was then stirred for 1 h at room temperature. 1 M glycine was added to a final concentration of 200 mM and stirred for an additional 1 h. Second, the peptide was reacted with maleimide-activated-ovalbumin (Imject™ Maleimide-Activated Ovalbumin, Thermo Fischer Scientific, Waltham, MA) according to the suppliers’ protocol. Briefly, the peptide was mixed with the activated ovalbumin at a 20:1 molar ratio in PBS and allowed to react at room temperature for 2 h. Each of the two peptide/OVA conjugates were dialyzed against PBS for 24 h at 4 °C, changing dialysis buffer every 6–8 h. The protein concentration was adjusted to 750 *µ*g/ml with PBS and then equal portions of the two peptide/OVA conjugates were mixed to then use as antigen. In a separate set of reactions, the peptide was coupled to keyhole limpet hemocyanin (KLH, Thermo Fischer Scientific, Waltham, MA) at a ratio of 200 molecules peptide per molecule KLH using the identical protocols (glutaraldehyde and maleimide-activated-KLH (Imject™ Maleimide-Activated KLH, Thermo Fischer Scientific, Waltham, MA) and equal portions of the resulting two peptide/KLH conjugates were mixed to use as antigen. Rabbits were immunized by Antibodies Inc. (Davis, CA) with the ITLN1 peptide/OVA antigen on days 1, 14, 21, and then boosted by immunization with ITLN1 peptide/KLH antigen on days 35 and 49 to produce the N-terminal polyclonal antisera (termed ITLN1^NTERM^). Preimmunization sera was collected and used as control antisera. All methods for these procedures were in accordance with relevant guidelines and regulations, and approved by the U.S. Department of Health and Human Services Public Health Service Animal Welfare Assurance Committee (Assurance ID D16-00576). Rabbit polyclonal antiserum generated by immunization with human ITLN1-derived synthetic peptides was described and characterized previously (termed ITLN^CONS^)^11,55^.

### Chicken anti-ITLN1 IgY

The ITLN1 protein sequence was analyzed using the online algorithm for design of peptide-directed antibodies provided by the NHLBI (https://hpcwebapps.cit.nih.gov/AbDesigner/)^56^. A 28 aa peptide (CTVGDRWSSQQGSKADYPEGDGNWANYN) was identified as an attractive antigen candidate. This peptide spanned the V108D polymorphic site, and was highly similar in sequence to human ITLN2 and C57BL/6 mouse Itln1 (27/28 identical residues to both of these orthologs). This peptide was synthetically prepared, purified and characterized^54^. In two separate reactions, the peptide was coupled to KLH, using the two chemical coupling strategies (glutaraldehyde and maleimide) and protocols, as described above. Each reaction product was dialyzed against PBS and protein concentration adjusted to 750 µg/ml. Equal portions of the two peptide/KLH conjugates were mixed to use as antigen. Leghorn chickens were given a primary inoculation with the KLH conjugate in complete Freund's adjuvant, followed by two booster inoculations at 3 and 6 weeks (with incomplete Freund's adjuvant) by Aves Labs, Inc. (Davis, CA). An enriched IgY fraction from egg yolk of immunized chickens (termed ITLN^CONS-FLOR^) was obtained by organic extraction de-lipidation, high-salt precipitation and then extensive dialysis against PBS.

### Bio-layer interferometry

Dissociation constants for recombinant ITLN1 with biotin-β-D-galactofuranose were determined using a bio-layer interferometry OctetRed instrument (ForteBio, Fremont, CA). Biotin-β-D-galactofuranose was diluted to 5 μM in PBS, pH 7.5, and loaded (120 s) onto Streptavidin biosensors (Forte Bio). The sensor was washed in PBS for 60 s, and then a baseline (60 s) established in (20 mM HEPES, pH 7.4, 150 mM sodium chloride, 10 mM calcium chloride, 0.1% Tween-20, 0.1% BSA). Recombinant ITLN1 was diluted to 50, 25, 10, 2.5, 1, 0.25, and 0.1 µg/mL in (20 mM HEPES, pH 7.4, 150 mM sodium chloride, 10 mM calcium chloride, 0.1% Tween-20, 0.1% BSA) and allowed to associate with the biotin-β-D-galactofuranose coated biosensor (200 s). Dissociation (200 s) was monitored by dipping biosensor in (20 mM HEPES, pH 7.4, 150 mM sodium chloride, 10 mM calcium chloride, 0.1% Tween-20, 0.1% BSA). Biosensors were regenerated between samples by triple-washing biosensors in 10 mM glycine pH 2.5 (5 s) followed by neutralization (5 s) in (20 mM HEPES, pH 7.4, 150 mM sodium chloride, 10 mM calcium chloride, 0.1% Tween-20, 0.1% BSA). The shake rate was set to 1000 rpm and binding assays were performed at room temperature. Data was plotted in Graphpad Prism software (version 9.0.0,Graphpad Software, San Diego, CA). The response at equilibrium (490 s) was plotted against concentration of ITLN1 and curves fit to a one-site total non-linear regression equation to determine equilibrium dissociation constants.

### Immunoblotting

Ileal non-CD and CD specimens, genotyped for *ITLN1* rs2274907 (V109D), were homogenized in assay buffer (150 mM NaCl, 1% Triton X-100, 0.5% sodium deoxycholate, 0.1% SDS, 50 mM Tris, pH 8.0) containing a cocktail (1:100) of protease inhibitors (Protease Inhibitor Cocktail III: 4-(2-aminoethyl)benzenesulfonyl fluoride (AEBSF, 1 mM), aprotinin (600 nM), bestatin (50 µM), E-64 (15 µM), leupeptin (20 µM),pepstatin (10 µM); Calbiochem/EMD Millipore, Burlington, MA). Protein concentration was determined by bicinchoninic acid (BCA) assay (Peirce, ThermoFischer Scientific). Following heating at 95 °C for 30 min (with or without 2.5% v/v 2-mercaptoethanol for reducing conditions), protein (25–50 μg/lane) was loaded to SDS–polyacrylamide gels (10% and 4–20% acrylamide) and electrophoresed for 40–50 min at 200 V, prior to wet transfer (Towbin Buffer: 25 mM Tris, 192 mM glycine, 20% v/v methanol, pH 8.3) to nitrocellulose membranes (0.2 μM pore, Bio-Rad Laboratories, Hercules, CA) at 350 mA for 60–80 min. Estimated molecular sizes of ITLN1 monomers and higher order oligomers were deduced using the MagicMark™ XP Western Protein Standard (ThermoFisher Scientific). Membranes were blocked in PBS-T containing 5% w/v skim milk for 30 min, and incubated with primary antibodies at 4 °C. Following overnight incubation, membranes were washed with PBS-T for 1 h, probed with donkey anti-rabbit IgG horseradish peroxidase (HRP) linked secondary antibody (GE Healthcare Amersham, Pittsburgh, PA) for 3 h at room temperature, rinsed in PBS-T, and visualized using Femto and ECL West chemiluminescent substrates (ThermoFisher Scientific). Chemiluminescent signal was detected using a Biospectrum AC Imaging System (UVP, Upland, CA).

### Immunohistochemistry (IHC)

Surgical tissue of human ileum and colon, fixed in Carnoy's fixative or HistoChoice™ Tissue Fixative (Sigma Aldrich, St. Louis, MO), were paraffin embedded and sectioned (4–5 μM) and mounted on X-tra™ positive-charged slides (Leica Biosystems, Buffalo Grove, IL). Sections were deparaffinized, rehydrated in gradient ethanol, H_2_O, and PBS and blocked with 5% donkey serum (in 1 × PBS) for 30 min prior to overnight incubation at 4 °C with antibody^51^. Following incubation, slides were washed, and probed with Vectastain ABC kit goat anti-rabbit/goat secondary antibodies (Vector Laboratories, Burlingame, CA), activated with 3,3-diaminobenzidine peroxidase (Vector Laboratories, Burlingame, CA), and counterstained in 0.4% light green (Sigma Aldrich, St. Louis, MO), 0.2% glacial acetic acid for 5 min prior to dehydration and mounting^51^. Samples were visualized using an Olympus BX51 microscope (Center Valley, PA) and images were processed using Adobe Creative Suite software (version 25, Adobe Inc., San Jose, CA).

### Fluorescence IHC

Specimen slides were prepared as before. Following overnight incubation with primary antibodies, samples were incubated with two secondary antibodies (goat anti-chicken IgY Alexa Flour Plus 647 [Thermo Fischer Scientific, Waltham, MA] and goat anti-rabbit IgG Alexa Flour 488 [Abcam, Cambridge MA]), rinsed, and then stained with DAPI using the TrueVIEW Autofluorescence Quenching Kit (Vector Laboratories, Burlingame, CA) as outlined by the manufacturer. Images were captured using a Leica SP8 STED 3 × microscope (Leica Microsystems Inc., Buffalo Grove, IL). Acquired Z-stacks were further processed to generate figure images using Fiji ImageJ software (version 1.0, https://imagej.net/Fiji)^57^.

### Statistical analysis

Statistical analysis was performed using Graphpad Prism software (version 9.0.0,Graphpad Software, San Diego, CA). Specific details are outlined in figure and table legends.

## Supplementary Information

Supplementary Information.
