# Supplementary material for: Human intelectin-1 (ITLN1) genetic variation and intestinal expression
Source: Sci Rep. 2021 Jun 18;11:12889. doi: 10.1038/s41598-021-92198-9 (PMC8213764; doi:10.1038/s41598-021-92198-9)
Supplement: Supplementary file 1 — Supplementary Information. [file 41598_2021_92198_MOESM1_ESM.pdf]

Supplementary Information

Human Intelectin-1 (ITLN1) Genetic Variation and Intestinal Expression

Eric B. Nonnecke*, Patricia A. Castillo, Amanda E. Dugan, Faisal Almalki, Mark A. Underwood, Carol A. De La Motte, Weirong Yuan, Wuyuan Lu, Bo Shen, Malin E. V. Johansson, Laura L. Kiessling, Edward J. Hollox, Bo Lönnerdal, and Charles L. Bevins*

Supplemental Figure 1. ITLN1 rs2274907 allele frequencies in African and European superpopulations as outlined by the 1000 Genomes Project Phase 3.

Supplemental Figure 2. ITLN1 mRNA and protein expression in gastrointestinal tissues assessed by qRT-PCR and immunohistochemistry, respectively.

Supplemental Table 1. ITLN1 rs2274907 (A/T, V109D) genotype analysis in IBD surgical specimens compared to controls.

Supplemental Table 2. Analysis of linkage disequilibrium on Chromosome 1 centromeric to *ITLN1*.

Supplemental Table 3. Quantitative trait loci analysis of whole blood using SNPs at the *ITLN1* locus identified by the NHGRI-EBI GWAS Catalog and NCBI-LitVar.

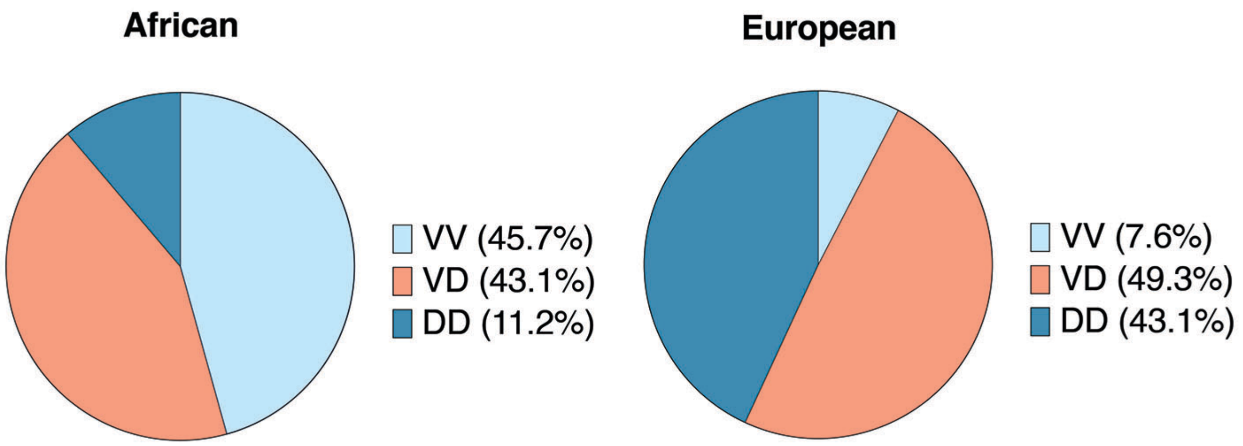

Supplemental Figure 1: (A) *ITLN1* rs2274907 frequency of AA (encoding VV), AT (encoding VD) and TT (encoding DD) genotypes in African and European superpopulations as outlined by the 1000 Genomes Project Phase 3 [31,32].

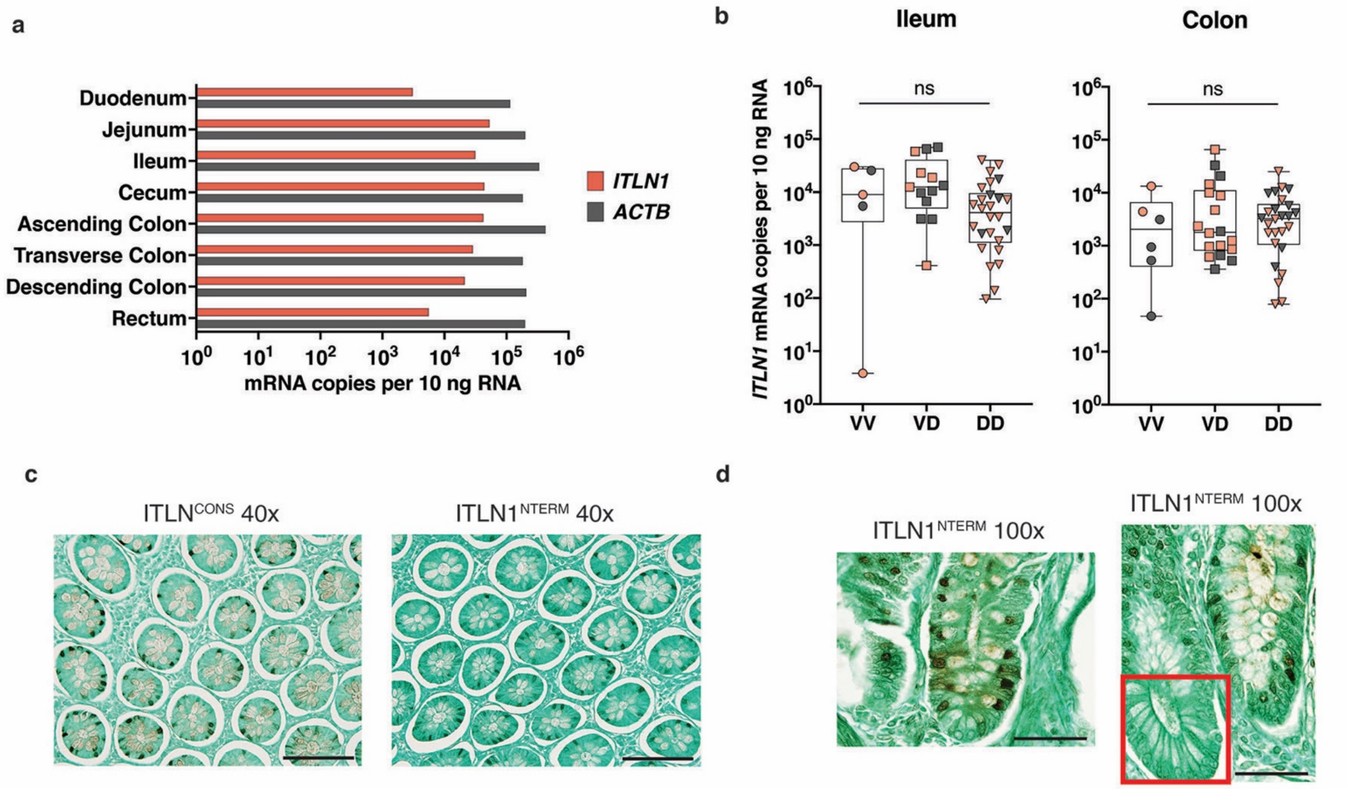

Supplemental Figure 2: (A) *ITLN1* and *ACTB* mRNA expression by qRT-PCR in commercially-obtained RNAs of human small intestine and colon. (B) *ITLN1* mRNA expression in ileum and colon specimens by genotype (rs2274907, V109D) in non-Crohn's disease (gray) and Crohn's disease (orange); ulcerative colitis specimens are excluded. Statistical analysis of *ITLN1* mRNA expression was performed by a Kruskal-Wallis test with Dunn's multiple comparisons. (C) Immunohistochemistry of human colon using ITLN conserved ($ITLN^{CONS}$, left) and ITLN1-specific ($ITLN1^{NTERM}$, right) antibodies. (D) Immunohistochemistry using the $ITLN1^{NTERM}$ antibody on human ileum demonstrating staining of goblet cells. Red box indicates collection of Paneth cells not stained with $ITLN1^{NTERM}$ antibody, but identified with the $ITLN^{CONS}$ and $ITLN2^{NTERM}$ antibodies (as in Figure 2G). Scale bars: 40x (100 μm) and 100x (50 μm).

Supplemental Table 1: ITLN1 rs2274907 (A/T, V109D) genotype analysis in IBD surgical specimens compared to controls.

	VV	*VD*	*DD*	*[AF, D]*	*p value*
Non-CD/UC	3 (4)	12 (2)	14 (1)	[68.9]	
CD Ileum	3	5	24	[82.8]	p = 0.09<br>OR: 2.17<br>CI: [0.95 – 4.95]
CD Colon	3	13 (1)	19	[72.8]	p = 0.697
UC Colon	0	17	13	[71.7]	p = 0.841
Non-CD/UC vs CD (All)					p = 0.21<br>OR: 1.56<br>CI: [0.75 - 3.03]

Due to major differences of allele frequency of African superpopulations that could artifactually influence statistical comparisons between IBD and non-IBD subgroups, the n = 8 specimens designated African/Black (numbers shown in parentheses) were omitted from analysis. Data were analyzed with a two-tailed $\chi 2$ with Fischer's exact test. OR = Odds ratio (Baptista-Pike), CI = 95% confidence interval, CD = Crohn's disease, UC = ulcerative colitis, AF = allele frequency.

	Location: Chromosome 1q23.3; GRCh38: CM000663.2 (Forward strand)	Consequence	Global Freq.	EUR SP Freq.	r^2: rs2274907	LD ($r^2 > 0.1$)	Allele correlated with rs2274907 (T)
CD244							
GWAS-Catalog							
rs4656940	1:160860478	A/G (intronic)	G: 33%	G: 19%	0.3646 (D' = 0.8487)	Yes	A
rs115868021	1:160841584	C/G (A129H)	G: 0%	G: 0%	0.0019	No	
rs569911	1:160828419	intergenic	T/C	C: 47%	0.0178	No	
NCBI-LitVar							
rs6682654	1:160839213	G/A (intronic)	A: 40%	A: 55%	0.0295	No	
rs3766379	1:160837925	T/C (intronic)	C: 56%	C: 56%	0.0188	No	
rs3766377	1:160830769	A/G (3' UTR)	G: 38%	G: 26%	0.0996	No	
rs3753389	1:160837363	T/C (intronic)	C: 57%	C: 45%	0.0192	No	
rs4656942	1:160861258	G/A (intronic)	A: 22%	A: 25%	0.1023 (D' = 0.8029)	Yes	A
rs76030314	1:160839020	T/C (V234I)	C:1%	C: 0%	N/A	No	
rs485618	1:160830690	C/T (3' UTR)	T: 37%	T: 53%	0.0178	No	
rs373331921	1:160841468	G/A (R133C)	A: 0%	A: 0%	N/A	No	
rs754083936	1:160834104	T/C (T303A)	C: 0%	C: 0%	N/A	No	
rs11265498	1:160858495	C/T (intronic)	T: 45%	T: 20%	0.3786 (D' = 0.8409)	Yes	C
rs749910003	1:160838965	C/T (R155K)	T: 0%	T: 0%	N/A	No	
LY9							
GWAS-Catalog							
rs540254	1:160797947	C/T (intronic)	T: 66%	T: 63%	0.1722 (D' = 0.7776)	Yes	C
rs3817407	1:160818341	T/C (intronic)	C: 36%	C: 42%	0.0927	No	
rs494091	1:160822102	T/C (intronic)	C: 63%	C: 45%	0.0217	No	
rs571841	1:160821621	C/T (intronic)	T: 63%	T: 45%	0.0209	No	
rs12128261	1:160792994	T/G (intergenic)	G: 20%	G: 23%	0.0962	No	
NCBI-LitVar							
rs509749	1:160823770	A/G (V602M)	G: 66%	G: 45%	0.0225	No	
rs560681	1:160816880	A/G (intronic)	G: 34%	G: 28%	0.1275 (D' = 0.3973)	Yes	A
rs512645	1:160828336	A/G (Reg.)	G: 57%	G: 47%	0.0171	No	
rs145664274	1:160818307	C/T (Stop)	T: 0%	T: 0%	N/A	No	
SLAMF7							
GWAS-Catalog							
rs489286	1:160752760	A/G (intronic)	G: 69%	G: 68%	0.0123	No	
rs35967351	1:160742014	A/T (intronic)	T: 19%	T: 30%	0.009	No	
rs11581248	1:160750284	C/T (intronic)	T: 4%	T: 13%	0.0817	No	
rs3766370	1:160741687	C/T (intronic)	T: 19%	T: 30%	0.0093	No	
rs12068654	1:160773959	T/G (intergenic)	G: 20%	G: 30%	0.0063	No	
rs983494	1:160734175	G/A (Reg. region)	A: 14%	A: 21%	0.004	No	
rs184772	1:160719355	G/A (intergenic)	A: 11%	A: 3%	0.0019	No	
NCBI-LitVar							
rs372671482	1:160749940	G/A (A166T)	A: 0%	A: 0%	N/A	No	
rs1232783379	1:160739302	A/G (No start)	G: 0%	G: 0%	N/A	No	
rs201990333	1:160750030	G/A (I62V)	A: 0%	A: 0%	N/A	No	
rs1418193833	1:160750012	G/T (D83Y)	N/A	N/A	N/A	No	
rs570311439	1:160752200	G/A (R115G)	A: 0%	A: 0%	0.005	No	
rs2295616	1:160740367	G/A (intronic)	A: 19%	A: 30%	0.0087	No	
rs758817538	1:160748217	A/G (K27E)	G: 0%	G: 0%	N/A	No	
rs117009784	1:160750053	A/C (R96S)	C: 2%	C: 0%	0.0105	No	
rs768547449	1:160750417	C/T (Stop)	N/A	N/A	N/A	No	
rs1364368414	1:160749822	G/A (Splice region)	N/A	N/A	N/A	No	
rs201003538	1:160748247	G/A (V37M)	A: 0%	A: 0%	0.0005	No	
rs1201685692	1:160752211	C/T (A206V)	N/A	N/A	N/A	No	
rs369009453	1:160739338	A/G (I13V)	G: 0%	G: 0%	N/A	No	
rs1287027241	1:160749955	G/C (P171A)	C: 0%	C: 0%	N/A	No	
rs1241870933	1:160750311	T/C (A219A)	N/A	N/A	N/A	No	
rs1440777816	1:160751358	G/C (E154D)	C: 0%	C: 0%	N/A	No	

rs755459128	1:160750102	C/G (L113V)	G: 0%	G: 0%	N/A	No	
rs1320318509	1:160751363	A/G (R263K)	G: 0%	G: 0%	N/A	No	
rs1209352977	1:160748422	T/C (L95P)	C: 0%	C: 0%	N/A	No	
rs767632168	1:160752226	C/G (C158S)	G: 0%	G: 0%	N/A	No	
rs978935748	1:160750306	G/A (A218T)	A: 0%	A: 0%	N/A	No	
rs757706994	1:160739316	A/C (P5P)	N/A	N/A	N/A	No	
rs17313034	1:160744058	T/C (intronic)	C: 15%	C: 29%	0.0071	No	
rs1314509893	1:160749863	A/G (N140S)	G: 0%	G: 0%	N/A	No	
rs141021747	1:160749994	T/A (W184R)	A: 0%	A: 0%	N/A	No	
rs1360254115	1:160749874	A/G (T144A)	N/A	N/A	N/A	No	
rs1238369872	1:160749961	G/A (E173K)	A: 0%	A: 0%	N/A	No	
rs576161510	1:160753131	C/T (T214M)	T: 0%	T: 0%	N/A	No	
rs1468582809	1:160750022	T/G (F193C)	N/A	N/A	N/A	No	

Supplemental Table 2: Analysis of linkage disequilibrium on Chromosome 1 centromeric to *ITLN1***.** Annotated SNPs 3' to *ITLN1* of *CD244*, *LY9*, and *SLAMF7* queried using the NHGRI-EBI GWAS catalog and NCBI-LitVar databases. Linkage disequilibrium (LD) to ITLN1 rs2274907 (C/T: V109D) was calculated using the LDpair Tool of the NIH-LDlink software, which references the 1000 Genomes Project (Phase 3) superpopulation (SP) data. An r^2 value of > 0.1 for correlated alleles was used as a threshold for LD, as suggested by LDPair (yellow highlight). The Ensembl database was utilized to determine allele frequencies for each SNP in global and European SP as well as regional consequences (e.g., nucleotide changes). Where noted, missense variants and putative functional changes (e.g., regulatory region) are indicated. The IBD-GWAS variant in intron 1 of CD244 (rs4656940: A/G) is highlighted in red. No additional variants in *CD244*, *LY9*, or *SLAMF7* have been identified by GWAS in relation to IBD or specifically Crohn's disease [31-35].

**Expression quantitative trait locus (eQTL) (Whole Blood)**	rs4656940 (A/G)	rs11265501 (G/A)	rs12058717 (C/T)	rs11578770 (C/T)	rs4656953 (C/T)	rs1333062 (T/G)	rs2274907 (A/T)	rs2274908 (G/A)	rs2274910 (T/C)	rs2297559 (G/A)	rs4656958 (A/G)
ITLN1	N/A	N/A	N/A	N/A	N/A	NES: 0.15 (0.000043)	NES: 0.15 (0.000053)	NES: 0.15 (0.000062)	N/A	NES: 0.21 (8.7e-8)	NES: 0.21 (4.8e-8)
CD244	NES: 0.15 (4.4e-15)	N/A	N/A	N/A	NES: -0.11 (7.3e-11)	NES: -0.11 (1.8e-11)	NES: -0.11 (1.7e-11)	NES: -0.11 (1.1e-11)	NES: -0.11 (6.9e-11)	NES: -0.094 (1.7e-8)	
**Splicing quantitative trait locus (sQTL) (Whole Blood)**	rs4656940 (A/G)	rs11265501 (G/A)	rs12058717 (C/T)	rs11578770 (C/T)	rs4656953 (C/T)	rs1333062 (T/G)	rs2274907 (A/T)	rs2274908 (G/A)	rs2274910 (T/C)	rs2297559 (G/A)	rs4656958 (A/G)
LY9	NES: -0.43 (1.2e-14)	NES: -0.41 (1.6e-14)	N/A	N/A	N/A	N/A	N/A	N/A	N/A	N/A	N/A
USF1	NES: -0.39 (4.4e-8)	NES: -0.35 (2.8e-7)	N/A	N/A	NES: 0.35 (2.6e-9)	NES: 0.34 (9.5e-9)	NES: 0.35 (4.4e-9)	NES: 0.35 (3.9e-9)	NES: 0.36 (1.2e-9)	NES: 0.37 (9.9e-10)	NES: 0.37 (1.0e-9)

Supplementary Table 3. Quantitative trait loci (QTL) analysis of whole blood using SNPs at the *ITLN1* locus identified by the NHGRI-EBI GWAS Catalog and NCBI-LitVar. The Genotype-Tissue Expression (GTEx) Portal was queried using eleven SNPs at the ITLN1 locus for expression QTLs (eQTLs) and splicing QTLs (sQTLs), which identified data entries for *ITLN1*, *CD244*, *LY9*, and *USF1*. The normalized effect size (NES) and associated p-values are indicated, unless not available (N/A). No significant data was generated for small intestine or colon QTLs from any SNP queries. No cell-type interaction eQTLs (ieQTLs) or cell-type interaction sQTLs (isQTLs) were identified by any SNP entry. The NES values reflect the variant allele (forward strand, as indicated in row 1).
